# Positive Affect Over Time and Emotion Regulation Strategies: Exploring Trajectories With Latent Growth Mixture Model Analysis

**DOI:** 10.3389/fpsyg.2020.01575

**Published:** 2020-07-21

**Authors:** Margherita Brondino, Daniela Raccanello, Roberto Burro, Margherita Pasini

**Affiliations:** Department of Human Science, University of Verona, Verona, Italy

**Keywords:** latent growth mixture modeling, trajectories, positive affect, emotion regulation strategies, longitudinal data

## Abstract

The influence of Positive Affect (PA) on people’s well-being and happiness and the related positive consequences on everyday life have been extensively described by positive psychology in the past decades. This study shows an application of Latent Growth Mixture Modeling (LGMM) to explore the existence of different trajectories of variation of PA over time, corresponding to different groups of people, and to observe the effect of emotion regulation strategies on these trajectories. We involved 108 undergraduates in a 1-week daily on-line survey, assessing their PA. We also measured their emotion regulation strategies before the survey. We identified three trajectories of PA over time: a constantly high PA profile, an increasing PA profile, and a decreasing PA profile. Considering emotion regulation strategies as covariates, reappraisal showed an effect on trajectories and class membership, whereas suppression regulation strategy did not.

## Introduction

Nowadays, the relevance of Positive Affect (PA) for many aspects of people’s life is well recognized, mainly on the basis of the positive psychology approach. Positive affect seems to influence people’s cognition and behaviors, to improve physical and mental health, and to promote good social relationships, with many consequences also on the quality of life and life satisfaction (see Lyubomirsky et al., 2005, for a review).

In this work, we focus on positive affect, defined as “the extent to which a person feels enthusiastic, active, and alert. High positive affect is a state of high energy, full concentration, and pleasurable engagement, whereas low positive affect is characterized by sadness and lethargy” (Watson et al., 1988, p. 1065). We refer to the theoretical framework distinguishing positive affect and negative affect (or activating and deactivating affect, according to more recent literature), being them both the structural dimensions (Burro, 2016) of affect more frequently characterizing English mood terms and the emotional dimensions underlying subjective well-being (Diener et al., 1985; Watson et al., 1988, 1999).

Positive affect is connected with many positive outcomes, such as psychological growth (e.g., Sheldon and Houser-Marko, 2001), mental health (e.g., Taylor and Brown, 1988; Tugade and Fredrickson, 2004), and physical health (e.g., Rasmussen et al., 2009). Positive affective states also contribute to an individual’s long term well-being, and they broaden individuals’ perspective making them more disposed to appreciate positive aspects in their lives, also influencing life satisfaction (Bryant, 2003; Quoidbach et al., 2010; Lyubomirsky and Layous, 2013; Farquharson and MacLeod, 2014; Douglass and Duffy, 2015).

In this paper, we focused on the study of changes of positive affect over time through an application of Latent Growth Mixture Modeling (LGMM), as a way to identify unobserved groupings in a longitudinal dataset permitting to capture temporal trends.

## Trajectories of Affect Over Time

Positive affect has been largely studied; however, only recently, attention has been paid to the description of its trajectories over time; this perspective should be more considered, given the fact that, as a state, positive affect fluctuates largely over time and across situations. Fluctuations in daily mood in adolescents, for instance, have been studied to identify distinct developmental trajectories, finding that adolescents with an increasing mood variability trajectory showed stable depressive and delinquency symptoms in early to middle adolescence compared with adolescents with a decreasing mood variability trajectory (Maciejewski et al., 2019). Patterns of change and stability in positive emotions, connected with physical education, assessed in secondary school students were found, and these patterns of variations were related with satisfaction of basic psychological needs and quality of motivation (Løvoll et al., 2019). Cece et al. (2019), using a three-wave design, found different emotional trajectories in athletes.

Some researches looking at changes in emotions along time are focused on weekly changes. Studies of variation of daily mood found an increasing of mood on the weekend relative to Monday through Thursday (Rossi and Rossi, 1977; Larsen and Kasimatis, 1990; Egloff et al., 1995; Reid et al., 2000; Reis et al., 2000; Helliwell and Wang, 2014; Young and Lim, 2014).

These findings suggest to deeply explore weekly changes in positive emotions, searching for different trajectories. We use a longitudinal design, assessing positive affect at seven time points, that is, seven days along one week, from Monday to Sunday. Longitudinal research studies with panel data are often applied to analyze processes of stability and change in individuals or groups. Working on this kind of data allows to explore individual differences and changes of patterns in variables over time. On the basis of the structural equation modeling methodology, it is possible to analyze longitudinal data using the latent class methods (Muthén, 2004; Green, 2014). This statistical approach models heterogeneity by classifying individuals into groups with similar patterns, called latent classes. In Growth Mixture Modeling (GMM), repeated measurements of observed variables are used as indicators of latent variables that describe specific characteristics of individuals’ changes. A special type of GMM is Latent Class Growth Analysis (LCGA) whereby all individual growth trajectories within a class are assumed to be homogeneous.

With this methodology, intercept and slope are considered two latent variables (also called random coefficients), which, respectively, represent the level of the studied variable when time is equal to zero, and the rate of change in the same variable over time. Given that few studies examined the trajectories of positive emotions over a week, no specific hypotheses were advanced regarding the number of trajectories, their characteristics (e.g., intercepts), or their evolution through time (e.g., linear and/or quadratic slopes).

These models also allow the inclusion of covariates (conditional model) as part of the same model of estimation of the trajectories (Nagin, 1999; Roeder et al., 1999; Muthén, 2004), evaluating the covariates’ impact on the longitudinal trajectory. In the present study, the conditional model evaluated the impact of emotion regulation strategies, assessed one week before the one-week daily positive affect assessment, on the trajectories.

## Emotion Regulation

Little is known about how emotion regulation strategies are associated with changes in positive affect in daily life, even if some emotion regulation strategies are shown to be related with changes in positive and negative affect (Brans et al., 2013; Gunaydin et al., 2016).

Emotion regulation strategies refer to the process through which people modify how they feel or express emotions they are experiencing (Gross, 1998, 2014, 2015; Gross and Thompson, 2007). This process can consist in the downregulation of negative emotions (that is, decreasing them) or in the upregulation of positive emotions (that is, increasing them) or in maintaining stable one’s own emotions. Upregulation of positive emotions has been shown to have a moderation effect on the relation between daily positive events and momentary happy mood (Jose et al., 2012). Furthermore, frequent use of positive upregulation strategies also seems to be associated with higher levels of happiness, life satisfaction, and positive emotions (Bryant, 2003; Quoidbach et al., 2010).

In the present study, we examined the relations between positive affect and emotion regulation strategies in terms of reappraisal and suppression emotion regulation strategies. Reappraisal is “a form of cognitive change that involves construing a potentially emotion-eliciting situation in a way that changes its emotional impact,” while suppression is “a form of response modulation that involves inhibiting ongoing emotion-expression behavior” (Gross and John, 2003, p. 349). Reappraisal and suppression strategies play a key role within the process of emotion regulation and are among the two emotion regulation strategies that are more investigated in the literature (Gross, 1998, 2014, 2015; Gross and Thompson, 2007). Taking as a framework Gross’ theoretical model, we know that people use them, respectively, when focusing on the antecedents of an emotion, for reappraisal, and when they focus on ways to modulate their responses, for suppression. These two strategies are particularly relevant in relation to positive affect, given empirical evidence documenting that people who frequently use reappraisal emotion regulation strategy experience more positive emotions, better relationships, a higher quality of life, and higher levels of well-being, compared to those who tend to prefer suppression (e.g., Kelley et al., 2019). For this reason, we hypothesize that reappraisal could affect positive emotion trajectories, whereas we expect that suppression does not.

## Aims

The main aim of this study is to show an application of LGMM, as a way to identify unobserved groupings in a longitudinal dataset. This technique was applied to the exploration of different trajectories of positive affect over a week, which corresponded to different profiles. Furthermore, we aimed at verifying whether the identified trajectories were affected by emotion regulation strategies, such as reappraisal and suppression.

## Method

### Participants

The participants were 108 undergraduate students (mean age: 22.2 years, *SD* = 6.2, range: 18–52 years; 84% female, 16% male) at the University of Verona, in Northern Italy, coming from a wide range of socio-economic status. They all took part to a larger micro-longitudinal study for which daily measures of students’ affect had been planned (e.g., Pasini et al., 2016; Raccanello et al., 2017, 2018; Burro et al., 2018). Students’ participation was voluntary, and all of them signed an informed consent form. The research was approved by the Ethics Committee of the Department of Human Sciences at University of Verona.

### Procedure

The study included two questionnaires. The first questionnaire was administered in group sessions in a pre-assessment phase, which took place 1 week before the beginning of the daily affect assessment. It included measures on emotion regulation strategies, as well as some demographic characteristics. The second questionnaire was administered through an on-line survey; it was presented daily for 1 week from Monday to Sunday. The participants received an e-mail message daily, at 10 a.m., in which they were reminded to answer to the on-line questionnaire between 6 p.m. and midnight. The on-line procedure permitted participants to answer with different kinds of devices. The use of different devices in psychological on-line research surveys has been proved to be connected with a good measurement quality and also with high participants’ compliance in a longitudinal design, with a low level of sample attrition (Pasini et al., 2016). This could be particularly true in affect assessment, because of the possibility, in contrast to what happens in laboratory studies, to record the construct of interest within the individual’s environment, increasing ecological validity (Shiffman et al., 2008). Furthermore, assessing affective states as they naturally occur, permits to avoid some biases connected with retrospective self-report methods, for instance, “peak,” and “recency” effects (Kahneman, 1999).

### Instruments

#### Daily Positive Affect

We used the 20-item Positive and Negative Affect Schedule (PANAS, Watson et al., 1988; Italian adaptation by Terracciano et al., 2003), considering only the subscale for the assessment of positive affective states. It consists of 10 items (e.g., active, enthusiastic, and excited), and participants rated on a 7-point scale the extent to which they had experienced each affect term (1 = *not at all* and 7 = *very much*), referring to the current day. This instrument was administered daily for 1 week.

#### Emotion Regulation Strategies

To assess emotion regulation strategies, we used the 10-item Emotion Regulation Questionnaire (ERQ, Gross and John, 2003; Italian adaptation by Balzarotti et al., 2010). Items had to be evaluated on a 7-point Likert scale (1 = *I completely disagree* and 7 = *I completely agree*). This scale assesses two different strategies: reappraisal (with six items, e.g., “I control my emotions by changing the way I think about the situation I’m in”) and expressive suppression (with four items, e.g., “I control my emotions by not expressing them”). The ERQ was administered in the pre-assessment group session.

#### Socio-Demographic Variables

We collected data on participants’ gender, age, and socio-economic status during the pre-assessment group session.

### Data Analyses

We carried out some preliminary analyses to assess the stability of the psychometric properties of the Positive Affect scale. This is an important preliminary step to properly conduct LCGA. First, we used a Confirmatory Factor Analysis (CFA) to evaluate the measurement model. The following combination of fit indices was used to evaluate the models (Brown and Moore, 2012; Kline, 2015): Chi-square degree of freedom ratio (*χ^2^*/*df*), the comparative fit index (CFI), the Tucker-Lewis index (TLI), the root-mean-square error of approximation (RMSEA), and the standardized root mean residual (SRMR), with *χ^2^*/*df* ≤ 2.0, CFI and TLI ≥ 0.90, RMSEA ≤ 0.08, and SRMR ≤ 0.06 as threshold values.

The second step concerned Measurement Invariance (MI), investigated in order to check the stability of the measurement model over the 7 days. Measurement Invariance (MI) analyses examined hypotheses on the similarity of the covariance structure over the 7 days, considering: (1) configural invariance, allowing all the parameters to be freely estimated; (2) metric invariance, requiring invariant factor loadings; (3) scalar invariance, also requiring invariant intercepts; and (4) uniqueness invariance, requiring invariant item uniqueness. Comparisons among models were based on differences in CFI and RMSEA, sample size independent; support for no changes in goodness of fit indexes requires a change in CFI and RMSEA less or equal than 0.010 and 0.015, respectively (Chen, 2007).

Then, we conducted LGMMs as a means of identifying growth trajectories of PA over seven time points, that is the 7 days of the on-line affect assessment, and to test predictors of the trajectories and of membership in these classes, using maximum-likelihood estimation to estimate class parameters (Muthén and Muthén, 2006). The analysis was performed in Mplus using the guidelines of Jung and Wickrama (2007).

First, as a preliminary analysis, we ran a single class latent growth curve model to define the best baseline model. We compared two growth curves: a first curve with the PA measures repeated on the 7 days as indicators and intercept and linear slope as higher-order latent factors, and a second one adding a quadratic parameter. Second, we specified a latent class model without covariates (unconditional). We evaluated the best-fitting model on the basis of the number of latent classes and the best-fitting parameters (linear vs. linear and quadratic). In order to compare the models, we used the information criteria and the fit indices. In addition, we followed the recommendations from the literature (e.g., Nylund et al., 2007), considering parsimony and interpretability as relevant criteria. We evaluated the Bayesian information criterion (BIC), and the Bootstrap Likelihood Ratio Test (B-LRT). We also assessed entropy values, to compare the degree of separation among the classes in the models, where scores closer to 1 highlight better fit of the data; the proportions for the latent classes (not less than 1% of total count in a class); and the posterior latent class probabilities (near to 1.00). After identifying the best unconditional model (free from covariates), we added into the model the two emotion regulation strategies as covariates (conditional model).

## Results

### The Stability of the Psychometric Properties of the Positive Affect Scale

Our results supported the goodness of fit of the hypothesized model analyzed running a CFA for each of the 7 days (see Supplementary Table S1). In the seven models, *χ^2^*/*df* ranged from 1.77 to 2.36, CFI from 0.92 to 0.95, RMSEA ranged from 0.087 to 0.114, and SRMR was always below 0.06, as recommended. The standardized loadings were all statistically significant at the 0.001 level. Therefore, our findings confirmed that the psychometric properties of the positive affect measure were acceptable across the 7 days.

MI analyses examined hypotheses on the similarity of the covariance structure across the different days. When we tested simultaneously the model over days, not imposing equality constraints between them (configural invariance), the goodness of fit of the models was confirmed. When all factor loadings were constrained to be equal (metric invariance), the models resulted invariant. When also the intercepts of the observed variables were constrained to be equal over days (scalar invariance), the models were invariant, as well as when factor loadings, intercepts, and residuals were constrained to be equal (uniqueness invariance). To sum up, the results of the sequence of gradually more restrictive tests of MI supported all the steps of invariance, confirming the stability of the measure of positive affect over the 7 days (fit indices of measurement invariance tests are reported in Supplementary Table S2).

### The Single Class Latent Growth Curve Model

At first, we conducted the analysis only with the estimate of the intercept. Then, we ran the model with the intercept and the slope parameters, and finally the model with the addition of the quadratic factor. The model with the estimation of the quadratic parameter did not converge, and the linear model, which considered intercept and slope, reported better fit indexes than the one with intercept only (see Table 1). Furthermore, examining the trajectories of the observed data, the linear growth curve seemed the more appropriate, and so it was retained. Moreover, estimates of variance related to the intercept and slope were significant, which justified an examination of interindividual differences in PA over time.

**TABLE 1:** Fit indexes, means, and variances of the parameters for the linear growth models (LGM).

Model	*χ^2^* (*df*)	CFI	RMSEA	SRMR	BIC	*M* intercept	Var intercept	*M* slope	Var slope
LGM (only intercept)	46.56 (26)	0.889	0.086	0.137	2074.72	3.75***	0.47***		
LGM (intercept, slope)	31.07 (23)	0.956	0.057	0.090	2073.28	3.87***	0.47***	−0.04	0.02*

*N* = 108. *df*, degree of freedom; CFI, comparative fix index; RMSEA, root mean square error of approximation; SRMR, standardized root mean square residual; BIC, Bayesian Information Criterion; M, mean; Var, variance. * *p* < 0.05, *** *p* < 0.001.

### Determining the Number of Classes

In the next step, we conducted the analyses to determine the number of latent classes. We compared progressive unconditional models from one to four classes, examining them on the basis of different elements. Model testing indicated that some parameters’ variance needed to be fixed to zero for the models to converge. We rejected the four-class model because the B-LRT highlighted that the three-class model was favored (*p* > 0.05). Even if for the two-class solution entropy was higher and BIC was slightly lower, the three-class solution seemed the more adequate on the basis of B-LRT (Table 2). Furthermore, the exploration of the trajectories confirmed the goodness of the three-class model, showing two trajectories with a constant level of PA along time, one high and one medium, and a decreasing PA trajectory.

**TABLE 2:** Information criteria and fit indexes for the unconditional GMM models.

Linear unconditional model (no covariates)						

Number of profiles	Parameters*	BIC	B-LRT *p*-value	Entropy	Number of subjects (%) in each class	Posterior probability estimate of class membership
2	16	2069.88	<0.05	0.77	70 (65%), 38 (35%)	0.95, 0.91
3	15	2072.80	<0.001	0.71	39 (36%), 37 (34%), 32 (30%)	0.81, 0.90, 0.88
4	20	2085.39	1.00	0.65	24 (22%), 19 (18%), 31 (29%), 34 (31%)	0.81, 0.73, 0.92, 0.73

BIC, Bayesian Information Criterion; B-LRT, Bootstrap Likelihood Ratio Test. * Intercept and slope variance fixed to zero in some cases.

### Adding the Covariates

**FIGURE 1:**
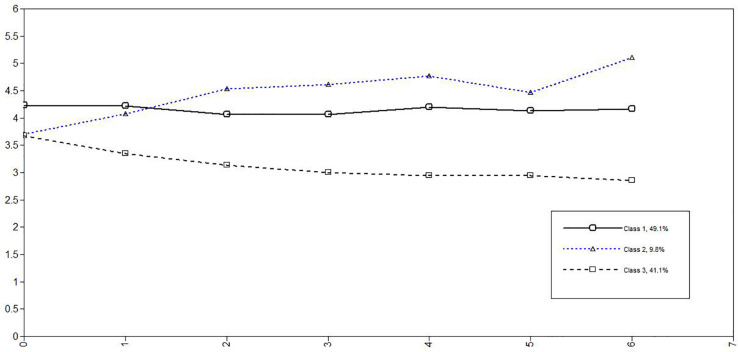
The three trajectories of Positive Affect along the week, from Monday (0) to Sunday (6), identified by the conditional model (observed means).

In the next step, the influence of covariates on trajectories and class membership was analyzed. Figure 1 shows the three PA trajectories along the week, from Monday (day 0) to Sunday (day 6) for the conditional model.

Table 3 shows the parameters’ estimates for the unconditional model and for the conditional one with reappraisal and suppression as covariates. Reappraisal regulation strategy showed an effect on trajectories for Constant High PA and Increasing PA trajectories, whereas suppression regulation strategy only showed an effect on intercept for Decreasing PA trajectory. About the effect on class membership, because the Constant High PA group comprises the largest number of participant (49), we decided to designate it as the reference class, and used logistic regressions to assess the degree to which the probability of being in the Constant High PA class was associated with each of the two covariates. Compared to the Constant High PA group, the coefficient of −0.74 (*p* = 0.037) for the Increasing PA class indicated that subjects were 0.74 times less likely to be assigned to the Increasing PA class. Relative to the Constant High PA class, the probabilities of latent class membership were significantly different by reappraisal regulation strategy. This means that Increasing PA class probability is lowered by high reappraisal values, relative to Constant High PA class. On the contrary, this regulation strategy did not show any effect on Decreasing PA class membership. No effect was found for suppression.

**TABLE 3:** Parameters’ estimates, information criteria, and fit indexes for the unconditional and conditional models.

	Trajectories of positive affect over time					
	
	Unconditional LGMM			Conditional LGMM^1^		
		
	Constant high PA	Constant medium PA	Decreasing PA	Constant high PA	Increasing PA	Decreasing PA
Mean intercept	4.69***	3.49***	3.41***	0.81	8.59***	4.41***
Mean slope	−0.02	0.04	−0.14*	0.28*	−1.15*	−0.45**
Intercept on reappraisal				0.79***	−0.47**	−0.07
Slope on reappraisal				−0.07*	0.37***	0.06
Intercept on suppression				−0.04	0.33	−0.21*
Slope on suppression				0.004	−0.03	0.03
Number of subjects (%) in each class	37 (34)	39 (36)	32 (30)	49 (49)	10 (10)	41 (41)
Posterior probability of class membership	0.91	0.81	0.88	0.90	0.92	0.90
Estimated parameters	15			32		
BIC	2072.80			1950.09		
B-LRT *p*-value	<0.001			0.08		
Entropy	0.71			0.78		

^1^In the conditional model, the variance of slope was constrained to 0. *p < 0.05, **p < 0.01, ***p < 0.001.

## Discussion

In the past decades, a large corpus of literature has amply documented how positive affect influences a variety of aspects within people’s everyday life, in terms of cognitive, behavioral, and also biological domains, in some cases identifying the nature of underlying mechanisms (Lyubomirsky et al., 2005). Positive psychology has shown its adaptive role for people’s health, describing the links between positive affect and both physical and psychological well-being in a variety of contexts (e.g., Taylor and Brown, 1988; Sheldon and Houser-Marko, 2001; Bryant, 2003; Tugade and Fredrickson, 2004; Rasmussen et al., 2009; Quoidbach et al., 2010; Lyubomirsky and Layous, 2013; Farquharson and MacLeod, 2014; Douglass and Duffy, 2015). However, only recently attention has been paid to the study of changes of affect over time, a highly relevant issue in light of the transient nature of affect, and of positive affect in particular, as a state.

Therefore, in order to extend current literature, we focused on the identification of trajectories of affect—specifically, of positive affect—examining micro-longitudinal data gathered within a larger project for which daily affect assessments had been planned (Pasini et al., 2016; Raccanello et al., 2017, 2018; Burro et al., 2018). We applied the LGMM analysis, a methodology that permitted to better understand the phenomenon of positive affect changes along a week, from Monday to Sunday. We identified three different trajectories which characterized three profiles of students: a profile with constant high levels of positive affect (Constant High PA), a profile showing an increasing trend of positive affect over the 7 days of assessment (Increasing PA), and a profile showing a decreasing trend (Decreasing PA).

According to Ryan et al. (2010), activities along the week, such as working or not working, as well as the weekday can have an effect on mood. People generally experience a higher level of positive emotions during weekends and non-working times. Furthermore, Fritz et al. (2010) found that relaxing activities carried on during the weekend, as well as the possibility to spend more time with family and friends, lead to more positive emotions. Our results seem to suggest that a deeper understanding of this effect is needed. In fact, in addition to the increasing PA profile, a constant high PA profile and a decreasing PA profile emerged. Mood variations across days during the week can result from many different factors, such as lifestyle, working condition, and social relationships. This allows us to suppose the stability of positive affect over time during a week for some people, whereas for other people, with the approaching of the weekend, the mood can change, improving in some cases, and getting worse in other cases.

We also examined how these profiles were affected by two among the most investigated emotion regulation strategies, reappraisal and suppression (Gross and John, 2003). From a theoretical perspective, this can shed some light on whether emotion regulation strategies play a role as protective or risk factors for people’s well-being (Diener, 2000). To do this, we estimated a conditional model, adding these two emotion regulation strategies as covariates. Results from the conditional model showed that the addition of reappraisal strategies affected the trajectories and, partially, the class membership, in particular decreasing the probability to be assigned to Increasing PA class, relative to the Constant High PA class. No effect was found for with the addition of suppression. In other terms, emotion regulation strategies played a role in characterizing the changes of positive affect over time in a differentiated way for reappraisal and suppression strategies. This result could be interpreted speculating that, on the whole, reappraisal could be responsible not only for feeling positively in a more intense way, but also for a higher stability of positive affect over time. However, further data should be considered to confirm this interpretation, considering for example the relations between emotion regulation strategies and profiles of negative affect over time.

Our study suffers from limitations related, for example, to the nature of self-report data. Furthermore, given the problems connected to the computational complexity and the reduced sample size, our results must be carefully considered. More research should be done to verify, for example, whether the three identified profiles could be generalized to other samples. Future researches should be also focused to check the stability of these results, looking at more than 1 week, and comparing the trajectories week by week. Despite these limitations, we think that, on the whole, we exemplified how the LGMM methodological approach could be used to identify and describe different trajectories of affect changes over time, extending what is currently known in the literature on positive affect. At an applied level, knowledge on such changes can be helpful when devising interventions aiming at favoring people’s well-being based on the awareness of their real inner states.

## Data Availability Statement

The datasets generated for this study are available on request to the corresponding author.

## Ethics Statement

The studies involving human participants were reviewed and approved by Ethics Committee of the Department of Human Sciences, University of Verona. The patients/participants provided their written informed consent to participate in this study.

## Author Contributions

MB and MP: project administration, conceptualization, data curation, formal analysis, investigation, data analysis, supervision, and writing original draft. DR: conceptualization, data curation, formal analysis, investigation, project administration, supervision, and writing original draft. RB: conceptualization, data curation, formal analysis, investigation, and supervision. All authors contributed to the article and approved the submitted version.

## Conflict of Interest

The authors declare that the research was conducted in the absence of any commercial or financial relationships that could be construed as a potential conflict of interest.
