# Supplementary material for: Positive Affect Over Time and Emotion Regulation Strategies: Exploring Trajectories With Latent Growth Mixture Model Analysis
Source: Front Psychol. 2020 Jul 21;11:1575. doi: 10.3389/fpsyg.2020.01575 (PMC7396512; doi:10.3389/fpsyg.2020.01575)
Supplement: Supplementary file 1 [file Table_1.docx]

Supplementary Material

**Table S1**

*Confirmatory Factor Analysis (CFA) fit indexes for the seven models, one for each day of the assessment of the Positive Affect (PA) scale*

| Model | *χ^2^* (*df* = 33) | *χ^2^*/df | CFI | TLI | RMSEA | SRMR |
| --- | --- | --- | --- | --- | --- | --- |
| Day 1 - PA | 60.41 | 1.83 | .936 | .912 | .089 | .054 |
| Day 2 - PA | 62.29 | 1.89 | .943 | .923 | .092 | .047 |
| Day 3 - PA | 58.32 | 1.77 | .953 | .936 | .087 | .049 |
| Day 4 - PA | 64.54 | 1.96 | .939 | .917 | .096 | .052 |
| Day 5 - PA | 77.79 | 2.36 | .919 | .890 | .114 | .060 |
| Day 6 - PA | 65.97 | 2.00 | .937 | .914 | .099 | .055 |
| Day 7 - PA | 70.04 | 2.12 | .935 | .911 | .105 | .049 |

*Note.* N = 108. df: Degree of freedom; CFI: Comparative Fix Index; TLI: Tucker-Lewis Index; RMSEA: Root Mean Square Error of Approximation; SRMR: Standardized Root Mean Square Residual.

**Table S2**

*Fit indexes of the models to test Measurement Invariance (MI) of the Positive Affect (PA) scale across the seven days*

| Model | *χ^2^* (df) | CFI | RMSEA | Δ CFI | Δ RMSEA |
| --- | --- | --- | --- | --- | --- |
| 1. Configural invariance | 455.09 (231) | .937 | .036 | - | - |
| 2. Metric invariance | 507.29 (285) | .937 | .032 | .000 | -.004 |
| 3. Scalar invariance | 576.42 (345) | .935 | .030 | -.002 | -.002 |
| 4. Uniqueness invariance | 663.51 (423) | .932 | .023 | -.003 | -.007 |

*Note.* N = 108. df: Degree of freedom; CFI: Comparative Fix Index; RMSEA: Root Mean Square Error of Approximation; Δ CFI: difference in CFI in comparison with the previous model (no change if ≤ 0.010); Δ RMSEA: difference in RMSEA in comparison with the previous model (no change if ≤ 0.015).
